# Prehospital COVID-19 patients discharged at the scene – an observational study

**DOI:** 10.1186/s12873-023-00915-6

**Published:** 2023-12-06

**Authors:** Kari Heinonen, Markku Kuisma, Heli Salmi, Tuukka Puolakka

**Affiliations:** 1 Department of Emergency Medicine & Services, Helsinki University Hospital and the University of Helsinki, P.O. Box 347, 00029 HUS Helsinki, Finland; 2 Department of Anaesthesiology & Intensive Care Medicine, Helsinki University Hospital and the University of Helsinki, Helsinki, Finland

**Keywords:** Emergency Medical Services, Covid-19, Prehospital, Non-conveyance

## Abstract

**Background:**

Emergency medical services (EMS) were the first point of contact for many COVID-19 patients during the pandemic. The aim of this study was to investigate whether the non-conveyance decision of a COVID-19 patient was more frequently associated with a new EMS call than direct ambulance transport to the hospital.

**Methods:**

All confirmed COVID-19 patients with an EMS call within 14 days of symptom onset were included in the study. Patients were compared based on their prehospital transport decision (transport vs. non-conveyance). The primary endpoint was a new EMS call within 10 days leading to ambulance transport.

**Results:**

A total of 1 286 patients met the study criteria; of these, 605 (47.0%) were male with a mean (standard deviation [SD]) age of 50.5 (SD 19.3) years. The most common dispatch codes were dyspnea in 656 (51.0%) and malaise in 364 (28.3%) calls. High-priority dispatch was used in 220 (17.1%) cases. After prehospital evaluation, 586 (45.6%) patients were discharged at the scene. Oxygen was given to 159 (12.4%) patients, of whom all but one were transported.

A new EMS call leading to ambulance transport was observed in 133 (10.3%) cases; of these, 40 (30.1%) were in the group primarily transported and 93 (69.9%) were among the patients who were primarily discharged at the scene (p<.001). There were no significant differences in past medical history, presence of abnormal vital signs, or total NEWS score. Supplemental oxygen was given to 33 (24.8%) patients; 3 (2.3%) patients received other medications.

**Conclusion:**

Nearly half of all prehospital COVID-19 patients could be discharged at the scene. Approximately every sixth of these had a new EMS call and ambulance transport within the following 10 days. No significant deterioration was seen among patients primarily discharged at the scene. EMS was able to safely adjust its performance during the first pandemic wave to avoid ED overcrowding.

**Supplementary Information:**

The online version contains supplementary material available at 10.1186/s12873-023-00915-6.

## Background

The changing role and the increasing workload of emergency medical services (EMS) and hospital emergency departments (ED) has led to the development of prehospital non-conveyance protocols, which allows ambulance crews to discharge patients at the scene instead of transport to hospital [1–3]. In Finland, up to 40% of patients seen by EMS are already routinely discharged at the scene after evaluation by EMS providers [4–6]. Non-conveyance practices elsewhere have been more conservative [7, 8].

The COVID-19 pandemic created a significant burden on both healthcare workers and systems worldwide [9, 10]. As many hospitals suffered from overcrowding, the role of EMS as the first point of contact with the healthcare system was emphasized [11–13]. Although the prehospital characteristics of COVID-19-patients have been described before [11, 14], studies on the potential and safety of prehospital non-conveyance in this context are lacking.

The aim of this study was to compare the characteristics of transported and non-conveyed COVID-19 patients and to determine whether non-conveyed patients had to recontact the EMS more often than patients primarily transported to hospital. The main outcome in the study was an EMS contact and ambulance transport within 10 days of the first EMS call. Additional points of interest were the reasons for EMS dispatch and transport, frequency of abnormal patient vital signs, and need for advanced life support procedures.

## Methods

### Study design

This was an observational cohort study covering all confirmed COVID-19 patients who used EMS after the World Health Organization (WHO) pandemic declaration onwards [15]. The study duration was 14.5 months between 11 March 2020 and 31 May 2021. Data collection was retrospective and based on electronic prehospital patient records. The study was conducted in accordance with the declaration of Helsinki. The study plan was approved by the institutional review board of the Helsinki University Hospital (HUS/247/2020) which evaluated that a separate ethical review board evaluation was not required due to the register-based nature of the study and the Finnish law on medical research (488/1999 and 984/2021). The study manuscript was prepared according to STROBE guidelines [16].

### Study setting

HUS is the largest academic hospital in Finland, serving a population of 1 700 000 in the Helsinki capital region. Emergency calls in the area are handled by emergency medical dispatchers working in regional emergency response centers. EMS in the region is governed by HUS and consists of basic life support and advanced life support ambulances each staffed by two emergency medical technicians or paramedics. EMS use a fully electronic patient case reporting (EPR) system (Merlot Medi®, CGI Inc, Montreal, Canada) for all patient records. In addition to hospital transport, the ambulance crews have the option to discharge a patient on the scene according to the non-conveyance protocol. Depending on the situation, the patient may be instructed to self-admit into an emergency department within the same day, to contact their own physician within a few days, or to stay at home and observe the situation. All non-conveyed patients are encouraged to recontact the emergency number if necessary. The protocol has been described in detail previously [5] and remained unchanged during the COVID-19 pandemic.

### Changes in EMS operation during the pandemic

During the first year of the pandemic, protection from vaccines was still incomplete and disease knowledge was limited. COVID-19 vaccinations at the HUS area began in late December 2020. By May 2021, 45% of the population in the HUS region had received their first vaccine and 9% were fully vaccinated [17, 18].

During this time, ambulance crews wore surgical facemasks and gloves during all patient contacts. The EPR system was modified to include structural forms to record both suspected COVID-19 and patient-reported laboratory-confirmed COVID-19 infection, which were recorded in all patient contacts. This information was registered in the EPR and relayed to the admitting hospital.

### Data collection

The data collection process was fully based on electronic patient records to minimize missing data. All EPR records after the WHO pandemic declaration of 11 March 2020 until 31 May 2021 were collected and screened for information of a laboratory-confirmed COVID-19 infection. The acquired prehospital reports were then further examined to evaluate the onset of COVID-19 symptoms [19, 20]. Patients with a COVID-19 related EMS call within 14 days of the symptom onset were included in the study. The study variables included patient age, sex, dispatch and transport code, dispatch and transport priority level, reason for possible non-conveyance, patient’s first vital sign measurements and the corresponding National Early Warning Score (NEWS), highest total NEWS, and reported symptoms. The lowest recorded value for blood oxygen saturation and the highest recorded values for body temperature were used. Studied interventions included supplemental oxygen, inhaled or intravenous medication, intravenous fluids, airway management, and use of mechanical or non-invasive ventilation (NIV). All values from the monitor defibrillator are sent to the EPR automatically via Bluetooth. For all manually entered parameters (e.g. temperature and blood glucose), the system will alert if a value with a clear error is entered. During the data analysis no values were excluded. As NEWS-score based cut-offs were used during analysis, error values were considered clearly abnormal.

### Study cohort

Patients were divided into groups based on whether their first registered EMS call led to ambulance transport or not. The primary outcome measure was a new EMS call and ambulance transport within 10 days of the first EMS call. The timeline was chosen to match the typical deterioration timeline in COVID-19 disease [21, 22]. Patients who were evaluated by the ambulance crews and did not require ambulance transport but were instructed to immediately admit to the ED by other means (e.g. taxi, private car) were categorized as “transported to hospital” in this study, as they too were evaluated at the hospital similarly to those transported. For the secondary analysis we examined all patients who had a new EMS contact that lead to transport within 10 days of the original EMS call. We aimed to identify, if those originally discharged at the scene by the EMS were in a worse condition when compared to those originally transported and discharged after a hospital evaluation.

### Statistical analysis

Data were analyzed using IBM SPSS statistical package (IBM, NY, USA). Pearson χ^2^ and Student’s t-test were used in comparison of groups when applicable. Significance was set at *p*<.05. Missing values were omitted from analysis.

## Results

**Fig. 1:**
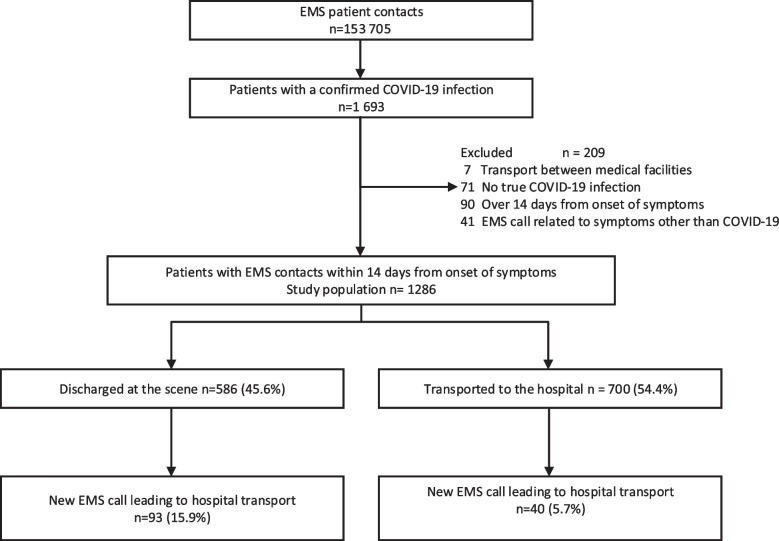
Description of the study population. EMS Emergency Medical Service

EMS had 153 705 patient contacts during the study period, of which 1286 (0.8%) met the study criteria (Fig. 1). Patients mean age (SD) was 50.5 (19.3) years and 47.0% were male. The most common dispatch codes were shortness of breath in 656 (51.0%) and malaise in 364 (28.3%) cases. Dispatch was made with high priority in 220 (17.1%) cases.

### COVID-19 patients discharged at the scene and transported to hospital

A total of 586 (45.6%) patients were discharged at the scene after evaluation by the ambulance crews; the remaining patients were primarily transported to the ED (Table 1). The discharged patients were younger (*p*<.001), less often had dyspnea, fatigue or fever (*p*<.001), and less frequently received >4 NEWS points (*p*<.001) than those who were primarily transported to the ED (Table 1). The vital parameters of both groups are available in Additional file 1. Administering supplemental oxygen resulted in ambulance transport in most cases (158 patients, 99.3%). However, only a few patients required inhaled or intravenous medication in either group. Only one patient required airway management on the scene due to sudden cardiac arrest. No other patients received NIV or mechanical ventilation.

**Table 1:** Prehospital COVID-19 patients who were discharged at the scene or transported to the hospital

**Variable**		**Discharged at the scene (*n*=586)**	**Transported to the hospital (*n*=700)**	***p*-value**
Age	*n*=1 286	46.59 (18.47)	53.79 (19.4)	<0.001
Male	*n*=1 286	280 (47.8%)	325 (46.4%)	0.628
Days from symptom onset		6 (4-8)	7 (5-9)	0.018
**Dispatch code**				
Dyspnea		291 (49.7%)	365 (52.1%)	0.375
Malaise		185 (31.6%)	179 (25.6%)	0.017
Chest pain		26 (4.4%)	52 (7.4%)	0.025
Nausea, vomiting or diarrhea		20 (3.4%)	28 (4.0%)	0.580
High priority used		69 (11.8%)	151 (21.6%)	<0.001
**COVID-19 symptoms**	***n*= 1286**			
Fever >38.0°C		170 (29.0%)	292 (41.7%)	<0.001
Dyspnea		265 (45.2%)	411 (58.7%)	<0.001
Fatigue		185 (31.6%)	354 (50.6%)	<0.001
Nausea, vomiting or diarrhea		126 (21.5%)	171 (24.4%)	0.215
Cough		177 (30.2%)	247 ( 35.3%)	0.054
Congestion or runny nose		76 (13.0%)	48 (6.9%)	<0.001
Loss of taste and smell		17 (2.9%)	21 (3.0%)	1
**Prehospital interventions**	***n*= 1286**			
Supplemental oxygen		1 (0.2%)	158 (22.6%)	<.001
Inhaled medication		1 (0.2%)	5 (0.7%)	0.229
Intravenous medication		2 (0.3%)	6 (0.9%)	0.303
EMS phycisian consulted		60 (10.2%)	108 (15.4%)	0.008
EMS phycisian on-scene		1 (0.2%)	3 (0.4%)	0.630
**National Early Warning Score (NEWS)**	***n*=1 249**			
0-2		381 (67.6%)	239 (34.9%)	<.001
3-4		138 (24.5%)	171 (25.0%)	0,84
5-6		38 (6.7%)	105 (15.3%)	<.001
>7		7 (1.2%)	170 (24.8%)	<.001
**Transport code**	***n*= 1286**			
Dyspnea		n/a	330 (47.1%)	n/a
Malaise		n/a	232 (33.1%)	n/a
Chest pain		n/a	29 (4.1%)	n/a
Nausea, vomiting or diarrhea		n/a	13 (1.9%)	n/a
High priority used		n/a	48 (6.9%)	n/a
Other means of transport used		n/a	42 (6.0%)	n/a
**Reason for non-conveyance**	***n*= 1286**			
Emergency care or transport not required		548 (93.5%)	n/a	n/a
Patient treated on the scene		20 (3.4%)	n/a	n/a
Patient refusal		16 (2.7%)	n/a	n/a
**New EMS call within 10 days**	***n*= 1286**			
New EMS call		138 (23.5%)	62 (8.9%)	<.001
New EMS call leading to ambulance transport		93 (15.9%)	40 (5.7%)	<.001

*EMS* Emergency medical services

Data are presented as mean (standard deviation), mean (standard deviation), median (interquartile range), or n (%) where applicable

The most common reason for non-conveyance was that neither emergency care nor ambulance transport was required. In some cases, the patient eventually refused to receive prehospital treatment or to be transported to the hospital. One patient was pronounced dead at the scene due to cardiac arrest. Like ambulance dispatch, dyspnea and malaise were the most common codes used for ambulance transport (Table 1). Only 48 (3.7%) patients were transported to hospital using high priority.

Pediatric patients formed a group of 24 patients, of whom 14 (58.3%), were discharged at the scene and 10 (41.7%) were transported to the hospital. One pediatric patient (4.2%) from those originally transported had new EMS call, but was not transported. No pediatric patients from those discharged at the scene had a new EMS contact.

### COVID-19 patients with a new EMS call leading to ambulance transport

A total of 200 (15.6%) COVID-19 patients had to contact EMS again after their first contact with EMS. This EMS call led to ambulance transport in 133 (66.5%) cases (Table 2). The recontact rate was over two times higher and the likelihood of ambulance transport nearly three times as high for patients who were primarily discharged at the scene than those who were primarily transported to the ED (*p*<.001).

**Table 2:** Patients with an EMS call requiring ambulance transport within 10 days of first EMS contact.

**Variable**		**Primarily discharged at the scene (*n*= 93)**	**Primarily transported to hospital (*n*= 40)**	***p*-value**
Age	*n*=113	56 (17.4)	49.6 (SD 18.7)	0.058
Male	*n*=113	48 (51.6%)	16 (40.0%)	0.219
Days from first EMS call	*n*=113	2 (1-4)	3 (2-5)	0.068
Days from symptom onset to new EMS call		9 (7-11)	8 (5-11)	0.171
**Medical history**	*n*=113			
Pulmonary disease		16 (17.2%)	5 (12.5%)	0.672
Hypertension		24 (25.8%)	11 (27.5%)	1
Hypercholesterolemia		20 (21.5%)	4 (10.0%)	0.181
Diabetes		17 (18.3%)	4 (10.0%)	0.346
Cardiac disease		6 (6.5%)	3 (7.5%)	1
**Dispatch code**	*n*=113			
Dyspnea		41 (44.1%)	23 (57.5%)	0.218
Malaise		34 (36.6%)	4 (10.0%)	0.004
Chest pain		4 (4.3%)	1 (2.5%)	1
Abdominal pain		2 (2.2%)	3 (7.5%)	0.160
High priority used		14 (15.1%)	9 (22.5%)	0.429
**Abnormal vital life functions**				
Respiratory rate ≤8 or ≥25 per minute	*n*=122	18 (20.7%)	8 (22.9%)	0.984
SpO2 ≤91%	*n*=130	36 (38.7%)	7 (18.9%)	0.050
Systolic BP <91 mmHg	*n*=117	0 (0%)	0 (0%)	n/a
Pulse rate ≤40 or ≥131 beats per minute	*n*=126	2 (2.2%)	2 (5.9%)	0.294
Glasgow Coma Score <9	*n*=124	0 (0%)	0 (0%)	n/a
Tympanic temperature ≤35 or ≥ 39.1 °C	*n*=129	17 (18.3%)	6 (15.0%)	0.804
Hypoglycemia (blood glucose < 4.0 mmol/l)	*n*=75	0 (0%)	0 (0%)	n/a
**National Early Warning Score (NEWS)**				
0-2		31 (33.3%)	17 (42.5%)	0.416
3-4		21 (22.6%)	6 (15.0%)	0,446
5-6		13 (14.0%)	10 (25.0%)	0.197
>7		28 (30.1%)	7 (17.5%)	0,194
**Prehospital interventions**	*n*=133			
Supplemental oxygen		28 (30.1%)	5 (12.5%)	0.053
Inhaled medication		1 (1.1%)	0 (0%)	1
Intravenous medication		1 (1.1%)	1 (2.5%)	0.513
EMS phycisian consulted		7 (7.5%)	6 (15.0%)	0.311
EMS phycisian on-scene		0 (0%)	1 (2.5%)	0.663
**Transport code**	*n*=133			
Malaise		44 (47.3%)	16 (40.0%)	0.557
Dyspnea		38 (40.9%)	17 (42.5%)	1
Chest pain		3 (3.2%)	1 (2.5%)	1
Nausea, vomiting or diarrhea		2 (2.2%)	1 (2.5%)	1
High priority used		6 (6.2%)	2 (5.0%)	1

Data are presented as mean (standard deviation), median (interquartile range), or n (%) where applicable

EMS=emergency medical services

National Early Warning score (NEWS) is highest total score given recorded during EMS call. Unmeasured vital functions are calculated as "0" by system

There were no significant differences in patient medical history between these groups (Table 2). The patients’ condition had not notably deteriorated, and abnormal vital signs were uncommon. Hypoxemia seemed to be more common in patients who were primarily discharged at the scene (*p*=.050). Apart from supplemental oxygen, few patients required any medication and none of the patients required airway management, NIV, or mechanical ventilation.

Among all the cases included in the study, one patient died on-scene on first EMS contact. He had cardiac arrest during transportation to hospital. While no deaths occurred in the population on follow-up EMS calls, a single death was found in the EMS setting within the 10-day period. A patient who refused transportation against medical advice on his first EMS call as well as on a follow-up EMS contact was found dead when the EMS arrived on the third call.

## Discussion

This is the first study reporting the use of a systematic, ambulance crew-initiated prehospital non-conveyance protocol in patients with a confirmed COVID-19 infection.

In this population-based cohort, nearly half of the prehospital COVID-19 patients could be discharged at the scene after EMS evaluation. The proportion of non-conveyance decisions in this patient group was over 50% higher than in the EMS calls from the same study setting in general [7]. This supports the finding by Satty and colleagues, in which the COVID-19 pandemic led to an increase in the overall non-conveyance rate [8]. At the time of primary EMS call, the patients discharged at the scene less often had COVID-19 symptoms and had significantly lower NEWS scores than patients who were selected for ambulance transport. An important dividing factor was the use of supplemental oxygen, as practically every patient requiring this was transported to the ED. The use of other medication or advanced life support procedures was rare.

The patients discharged at the scene had to contact EMS again within the following 10 days more than twice as often than those primarily transported to the ED. The observed recontact rate in patients discharged at the scene (23.5%) was nearly four times as high as previously reported by Paulin and colleagues in the general prehospital population [3]. Typically, EMS were contacted again 2 or 3 days after the first EMS call when the patient’s symptoms had been developing for more than a week in total. This finding is consistent with pattern of patient deterioration commonly observed in COVID-19 [21, 22].

Among the patients originally discharged at the scene, a new EMS call led to transport in almost 70% of cases. At the time of discharge at the scene, nearly 90% of these patients had a low-risk NEWS score of 0-4. At the time of the new EMS call, this proportion decreased to <60%. Nevertheless, the use of medication and prehospital procedures was uncommon apart from supplemental oxygen.

The data showed seven patients discharged at the scene with a recorded highest NEWS score of 7 of more. In a case-by-case evaluation, these included two patients who refused transportation against medical advice and a nursing home patient that was seen to be terminal and was given proper palliative care at the present residence. The other four patients clearly improved after initial assessment and the final NEWS scores recorded were between 2 and 4. Only one of these patients had a new EMS call, which ended in a non-urgent transport.

Although the recontact rate of prehospital COVID-19 patients discharged at the scene was high, most of these patients did not have to recontact the EMS system. Based on earlier data, the number of patients who contacted the ED afterwards was <5% [3]. These patients can also use non-urgent pathways, such as public healthcare centers and private outpatient clinics. Considering that the patients’ vital signs were rarely abnormal, NEWS scores remained low, and the need for ALS procedures was virtually non-existent, prehospital discharge of COVID-19 patients appears to be a safe and effective method to decrease the burden of hospital EDs.

Based on the data in this study we have found that the patients that later deteriorate and require hospital transport cannot be identified from other patients on initial EMS contact. A significant number of new EMS contacts are seen also among those initially transported. Based on our data, no clear cut-offs or scores for vital parameters can be given. With over 45% of patients not requiring transport only six patients were later transported to the hospital with high priority, a percentage similar to that seen in those initially transported. We feel that the non-conveyance criteria used in all EMS patients may also be used on COVID-19 patients when the criteria for non-conveyance are met. A list of the non-conveyance criteria used by the Helsinki EMS system is available in Additional file 2.

The strength of this study was the consecutive patient sample based on the EPR system, which provided conclusive prehospital data (including vital signs) that allowed reconstruction of the patients’ NEWS scores. The system also allowed the ambulance crews to register the patients’ COVID-19 status (laboratory-verified or not), which provided the foundation for this study. The study was limited by the single-system sample and the relatively short duration of data collection corresponding to the natural progression of the pandemic. Confirmation of COVID-19 infection relied on the information in the prehospital patient report and was not double-checked from laboratory databases. The investigators neither had access to the patients’ hospital records which could have provided more detailed information of their risk factors for COVID-19 as well as condition and length of hospital stay. Some patients with worsening condition may have contacted the ED directly and not reactivate the EMS.

## Conclusion

Nearly half of all prehospital COVID-19 patients could be discharged at the scene. Approximately every sixth of these patients had a new EMS call and ambulance transport within the following 10 days. However, abnormal vital signs or the need for advanced life support procedures was rare and no significant deterioration was seen. EMS could safely adjust its´s performance during the first pandemic wave to avoid ED overcrowding.

### Supplementary Information

**Additional file 1.** Vital parameters of COVID-19 patients who were discharged at the scene or transported to the hospital. The National Early Warning Score points for the vital parameters of prehospital patients with confirmed COVID-19. Patients under 16 years of age were not included from this data, as a separate score is used for pediatric patients.

**Additional file 2.** Non-conveyance criteria used by the Helsinki Emergency Medical Services.

## Data Availability

The datasets generated and/or analyzed during the current study are not publicly available due to the research approval not allowing data distribution to third parties. These datasets are available from the corresponding author on appropriate request with required research approvals.

## Abbreviations

ALS: Advanced life support
COVID-19: Coronavirus disease 2019
ED: Emergency department
EMS: Emergency medical services
EPR: Electronic patient case reporting system
GCS: Glasgow coma score
HUS: Helsinki University Hospital
IQR: Interquartile range
NEWS: National early warning score
NIV: Non-invasive ventilation
SD: Standard deviation
WHO: World Health Organization
