# Supplementary material for: Prehospital COVID-19 patients discharged at the scene – an observational study
Source: BMC Emerg Med. 2023 Dec 6;23:145. doi: 10.1186/s12873-023-00915-6 (PMC10701921; doi:10.1186/s12873-023-00915-6)
Supplement: Supplementary file 1 — Additional file 1. Vital parameters of COVID-19 patients who were discharged at the scene or transported to the hospital. The National Early Warning Score points for the vital parameters of prehospital patients with confirmed COVID-19. Patients under 16 years of age were not included from this data, as a separate score is used for pediatric patients. [file 12873_2023_915_MOESM1_ESM.pdf]

Additional file 1

Title of data:
Vital parameters of COVID-19 patients who were discharged at the scene or transported to the hospital.

Description of data:
The National Early Warning Score points for the vital parameters of prehospital patients with confirmed COVID-19. Patients under 16 years of age were not included from this data, as a separate score is used for pediatric patients.

Additional File 1: Vital parameters of COVID-19 patients who were discharged at the scene or transported to the hospital.

Variable	Discharged at the scene (n=586)	Transported to the hospital (n=700)	p-value
First respiratory rate (n=1191)			
NEWS 0	498 (92.1%)	404 (62.2%)	<0.001
NEWS1	0 (0%)	0 (0%)	-
NEWS 2-3	43 (7.9%)	246 (37.8%)	<0.001
Lowest blood oxygen saturation (n=1229)			
NEWS 0	413 (74.5%)	296 (43.9%)	<0.001
NEWS1	108 (19.5%)	121 (17.9%)	0.482
NEWS 2-3	33 (6.0%)	259 (38.2%)	<0.001
First Systolic Blood pressure (n= 1 102)			
NEWS 0	467 (92.3%)	520 (87.2%)	0.006
NEWS1	37 (7.3%)	46 (7.7%)	0.799
NEWS 2-3	2 (0.4%)	30 (5.0%)	<0.001
First masured heart rate			
NEWS 0	290 (52.6%)	277 (41.3%)	<0.001
NEWS1	202 (36.7%	255 (38.1%)	0.615
NEWS 2-3	59 (10.5%)	138 (20.6%)	<0.001
First Glasgow Coma Score <9 (n=1169)			
NEWS 0	530 (99.8%)	621 (97.3%)	<0.001
NEWS 2-3	1 (0.2%)	6 (0.9%)	0.135
Highest tympanic temperature (n=1 222)			
NEWS 0	355 (64.3%)	358 (53.4%)	<0.001
NEWS1	155 (28.1%)	210 (31.3%)	0.215
NEWS 2-3	42 (7.6%)	102 (15.2%)	<0.001

National Early Warning Score (NEWS) points for vital parameters for prehospital COVID-19 patients at the scene or transported to the hospital.
Patients unders 16 year of age are not included
