# Supplementary material for: Prehospital COVID-19 patients discharged at the scene – an observational study
Source: BMC Emerg Med. 2023 Dec 6;23:145. doi: 10.1186/s12873-023-00915-6 (PMC10701921; doi:10.1186/s12873-023-00915-6)
Supplement: Supplementary file 2 — Additional file 2. Non-conveyance criteria used by the Helsinki Emergency Medical Services. [file 12873_2023_915_MOESM2_ESM.pdf]

Additional file 2: Non-conveyance criteria used by the Helsinki Emergency Medical Services

1. Reason that leads to the EMS call is clear. The symptoms have passed or lessened, and the situation does not require further investigation.
2. Patient is able to take care of himself or appropriate supervision is available (e.g. family member or nurse present)
3. All appropriate examinations have been made or evaluated to be unnecessary (e.g. minor injury in a patient clearly in good condition)
4. No vital parameter is clearly abnormal and an explanation is known for slightly abnormal vital parameters. (i.e. tachycardia in a patient with fever)
5. EMS personnel have evaluated that the patient will not benefit from transport and no other reason supporting patient transport is present.
6. If above criteria are not met or situation remains unclear, EMS personnel are directed to consult the EMS physician.
7. If patient refuses transport, mental state must be registered to evaluate if patient is competent to make informed decision. In all unclear cases the EMS physician must be consulted to evaluate need for transport against the will of the patient
8. In all elderly nursing home patients, patients with multiple comorbidities or patients with DNAR decisions, evaluation of benefits of transport must be made compared to the care available at present location.
9. If a patient is not transported, the non-conveyance checklist should be filled and the patient report printed out as written information for the patient.
10. Patient is given written instructions for home care with details to make new emergency call, if their condition weakens.
